# Seeds of Discomfort: An Unusual Case of Pediatric Abdominal Pain

**DOI:** 10.7759/cureus.50625

**Published:** 2023-12-16

**Authors:** Katherine O Salada, Emily Jacobson

**Affiliations:** 1 Division of Pediatric Hospital Medicine, Department of Pediatrics, C.S. Mott Children’s Hospital, University of Michigan, Ann Arbor, USA; 2 Division of Hospital Medicine, Department of Internal Medicine, Michigan Medicine, Ann Arbor, USA

**Keywords:** pediatric hospital medicine, unexplained abdominal pain, sigmoid volvulus, stercoral colitis, seed bezoar

## Abstract

This case describes a seven-year-old healthy boy who presented with seven days of abdominal pain, small-volume liquid stools, tenesmus, fevers, and dehydration after consuming an unknown amount of shelled watermelon seeds. He was ultimately found to have a large rectal seed bezoar that caused irritation, resulting in stercoral colitis with rectal inflammation. He was additionally found to have sigmoid volvulus during one of his disimpactions, which was also likely secondary to his rectal seed bezoar. This case uniquely highlights the importance of maintaining an index of suspicion for rectal seed bezoars, discusses previously unreported pediatric complications of rectal seed bezoars, including stercoral colitis and sigmoid volvulus, and addresses the management of this rare presentation.

## Introduction

Abdominal pain is a common chief concern in pediatric medicine, with a broad differential including constipation with overflow stool and infectious gastroenteritis when fever and emesis are also present. In this report, we present a case of abdominal pain originally misdiagnosed both clinically and on imaging as simple constipation with stool impaction. After the patient failed to improve and a further history of preceding consumption of a large quantity of shelled watermelon seeds was obtained, the differential was broadened and further workup was pursued, leading to the correct diagnosis. Rectal seed bezoars, sterocoral colitis, and sigmoid volvulus are three rare pathologies that can each cause pediatric abdominal pain, all of which are uniquely seen together in this single pediatric case.

## Case presentation

A seven-year-old healthy boy presented to the emergency department with seven days of tenesmus, abdominal pain, and dehydration. Stools were described as brown, small-volume, liquid, and “foul-smelling.” He endorsed waking to stool overnight, the sensation of incomplete emptying when stooling, and stooling every one to two hours. Additionally, he reported tenesmus, fecal incontinence, rectal pain, and generalized abdominal pain. The family reported a small amount of bright red blood in his stool on the day of presentation and that his anus “looked wider than previously,” but otherwise denied hematochezia and melena. He did not have a history of constipation.

He ate an unknown amount of shelled watermelon seeds in the weeks leading up to symptom onset, with the family noting intermittent full shells in his stools. The family otherwise endorsed a normal and unrestricted diet history. Three weeks prior to presentation, he was treated with a course of amoxicillin for streptococcal pharyngitis while vacationing in Mexico. There had been no emesis, weight loss, or fevers.

**Figure 1:**
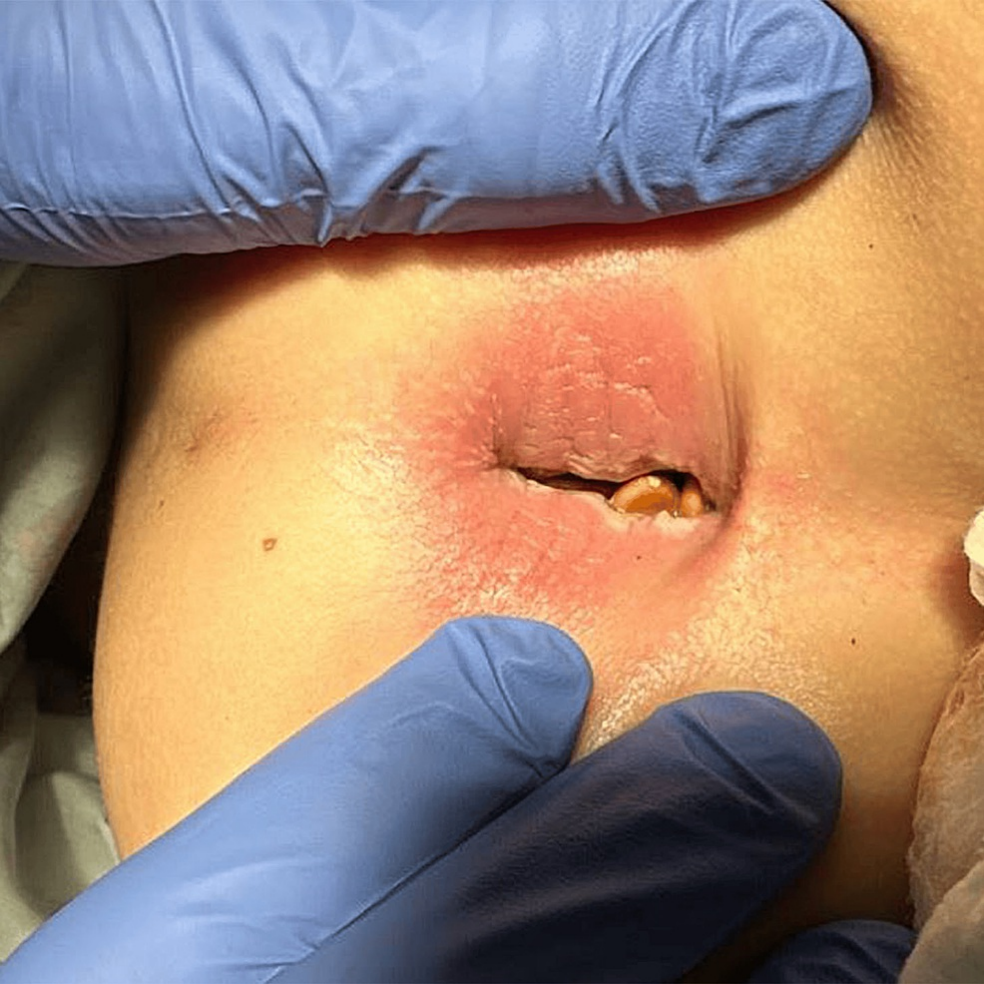
Rectal exam on admission showing large anal fissure

**Figure 2:**
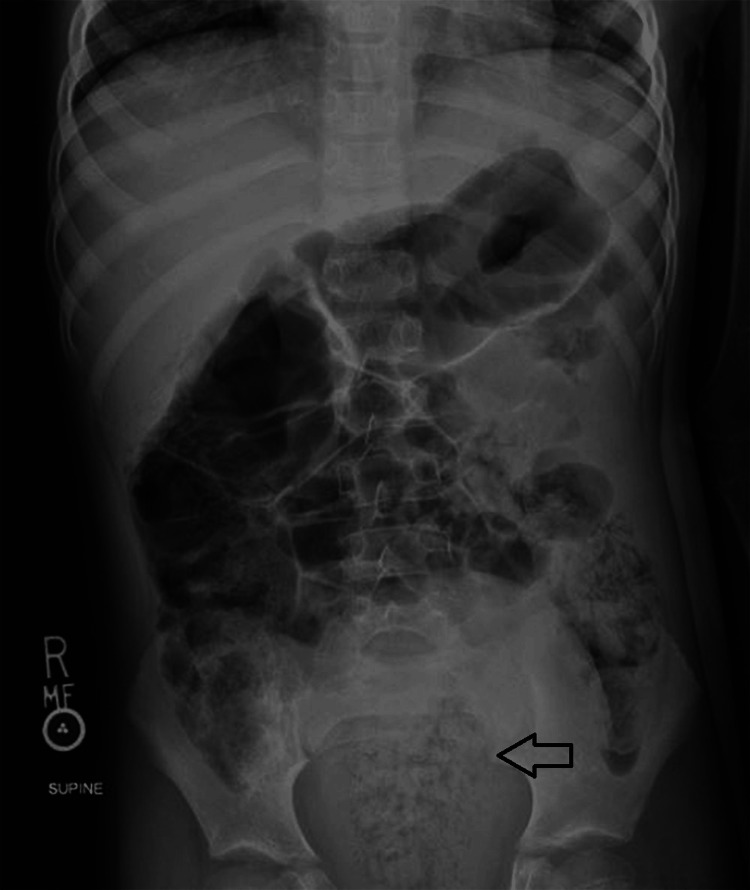
Abdominal X-ray on admission demonstrating moderate stool burden in the colon with a large stool burden in the rectal vault of 1.89 in (4.8 cm) that is identified by the black arrow

On arrival, he was febrile to 38.2°C with a pulse rate of 117 beats/minute, blood pressure of 111/69 mmHg, and respiratory rate of 28 breaths/minute. The physical exam was notable for generalized abdominal tenderness and fullness, without abdominal masses or peritoneal signs, and a large anal fissure (Figure 1). His labs showed a leukocytosis of 14,300 WBCs per mL (14.3 × 109/L), CRP 18.2 mg/dL (182 mg/L), and ESR 33 mm, with an otherwise normal complete blood count and comprehensive metabolic panel. A multiplex PCR panel for gastrointestinal pathogens and stool microscopy for ova and parasites were negative. Fecal calprotectin testing was elevated (2,314 mg/kg). An abdominal X-ray showed a non-obstructive bowel gas pattern with a large amount of formed stool in the rectal vault (Figure 2). Pediatric surgery was consulted and medical management was recommended for his anal fissure. He was admitted to the general Pediatric Hospital Medicine service for bowel cleanout, intravenous fluid hydration, and pain management.

**Figure 3:**
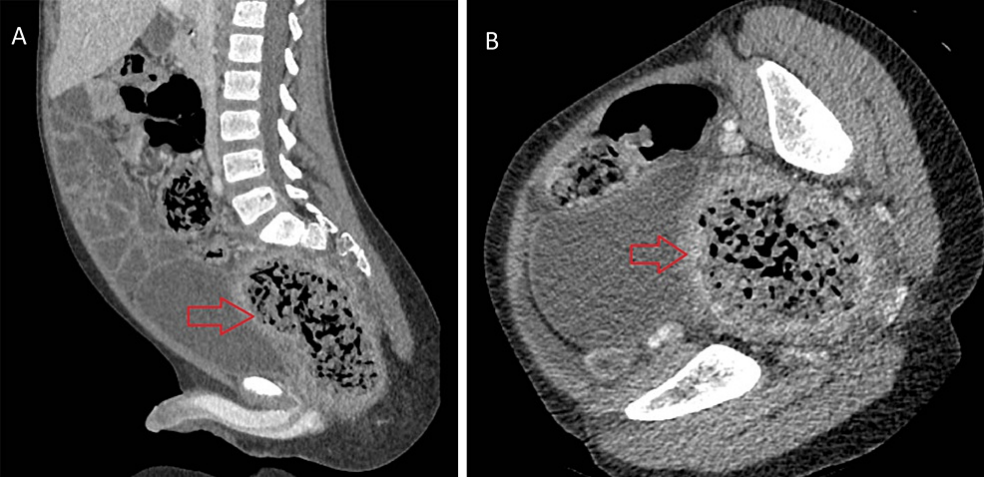
CT images showing large rectal fecalith in rectum measuring 2.6 in (6.6 cm) with mild circumferential rectal wall thickening and enhancement, with perirectal and presacral fat stranding consistent with stercoral colitis; key findings are marked by the red arrows

**Figure 4:**
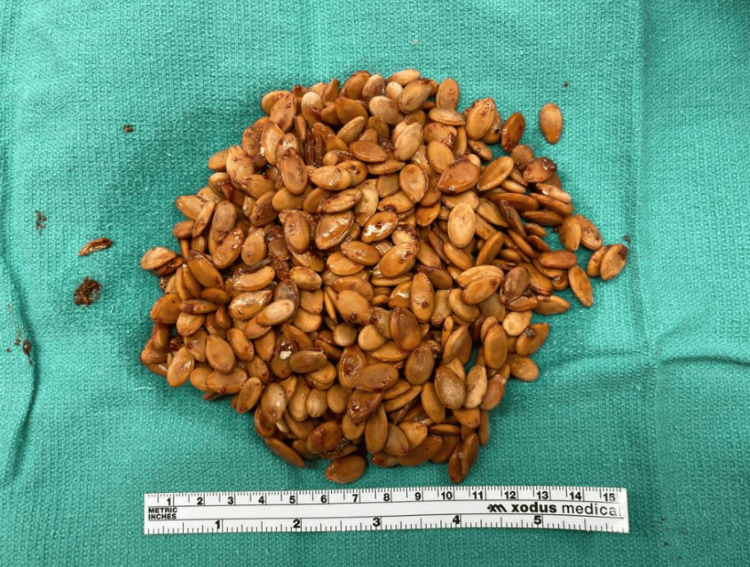
Watermelon seeds removed during one of the patient’s rectal disimpactions

**Figure 5:**
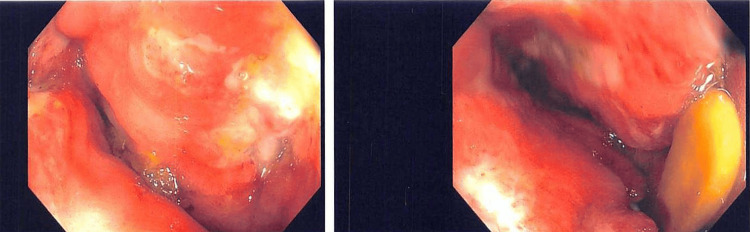
Sigmoidoscopy noting moderate inflammation and ulceration in the rectum

Over the next 24 hours, he developed progressively worsening abdominal pain. A CT abdomen/pelvis was obtained on day two and showed moderate fecal loading and a large rectal fecalith with mild circumferential rectal wall thickening and enhancement, with perirectal and presacral fat stranding (Figure 3). Given his fever, worsening pain and exam, and imaging findings, the patient was started on ceftriaxone and metronidazole, and surgery was re-engaged. He underwent operative fecal disimpaction on day three, where a large seed bezoar (Figure 4) was found and removed. Contrast enema post-op showed concern for residual seeds; thus, he was started on nasogastric GoLytely cleanout on day three. Despite passing seeds with GoLytely, he continued to have significant abdominal and rectal pain that was not relieved with intravenous opiates and topical lidocaine and nifedipine applied to his anal fissure. He underwent a second sedated disimpaction on day four, at which point a sigmoid volvulus was noted and de-torsed endoscopically. During the case, he was noted to have moderate inflammation and ulceration in the rectum (Figure 5). Antibiotics were discontinued on day four due to improving clinical status and a lower concern for infectious complications. Finally, he underwent a third fecal disimpaction and was discharged on hospital day seven.

Given the atypical presentation and significant rectal inflammation on endoscopy, underlying pathology such as inflammatory bowel disease was considered as a potential predisposing factor. Although prior literature notes healthy patients without risk factors for seed bezoars (besides occasional constipation), underlying colonic inflammation could predispose to seeds getting stuck and the development of a seed bezoar. Consideration of repeat outpatient endoscopy, colonoscopy, and magnetic resonance enterography three months after the acute episode was discussed to ensure his inflammation had resolved. Given that the patient had no further gastrointestinal symptoms, experienced resolution of his anal fissure within weeks, and continued to gain weight, further work-up was deferred.

## Discussion

The leading diagnosis for this patient is that the large volume of consumed seeds led to the development of a rectal seed bezoar that was misidentified as a fecalith on imaging; this seed bezoar caused irritation, resulting in stercoral colitis with rectal inflammation. He was additionally found to have sigmoid volvulus during one of his rectal disimpactions, which was also likely secondary to his rectal seed bezoar. Stercoral colitis is rarely documented outside of adults, with few case reports in pediatrics, and there are no cases of seed bezoar causing volvulus previously published in the literature.

Bezoars are retained, indigestible material that can accumulate within the gastrointestinal tract. Bezoars are classified by composition, with seed bezoars being a subset of the most common type of bezoar, phytobezoars (fruit and vegetable material) [1]. While gastric bezoars are more common overall, seed bezoars are most often found in the rectum [2]. Compared to gastric bezoars, rectal seed bezoars remain infrequently documented in the literature, particularly in pediatric patients [3-5]. In the cases described, watermelon seeds, followed by prickly pear and sunflower seeds, are most often reported [3]. It is speculated that the size of the seeds allows passage through the stomach and small intestine, leading to accumulation in the colon [2]. For this reason, children with rectal seed bezoars generally have no underlying predisposing conditions [3], which contrasts with the high prevalence of underlying gastrointestinal dysmotility noted in those with gastric bezoars [6]. Similar to our patient, the most common presenting symptoms of rectal seed bezoars are constipation followed by abdominal/rectal pain [2]. The typical treatment includes fecal disimpaction, either manually or surgically, with less success with chemical dissolution (e.g., Coca-Cola) compared to fiber bezoars [7]. When reported, cases of rectal seed bezoars typically focus on diagnosis and treatment, with few documented complications.

The patient’s hospital course was notable for complications of both stercoral colitis and sigmoid volvulus secondary to his large rectal seed bezoar. Stercoral colitis is an inflammatory colitis thought to be secondary to pressure necrosis from a fecal mass leading to hypoperfusion. The prevalence is highest in elderly adults with only a few case reports published in the pediatric literature [8-10]. It is most often reported as a complication of constipation, with no case reports of stercoral colitis secondary to seed bezoar. With case reports of rectal ulceration in adults with seed bezoars, it is possibly underdiagnosed in children given the rarity in pediatrics. Given the non-specific symptoms of abdominal pain, distension, and other gastrointestinal symptoms, a high index of suspicion is needed to make the diagnosis. CT imaging is often required for diagnosis, with the most common imaging findings in adults including large stool burden, bowel wall thickening/inflammation/mucosal hyperenhancement, and fat stranding [11]. Prompt recognition of this condition and early disimpaction are important given the risks of peritonitis, bowel perforation, and sepsis if left untreated.

Similar to stercoral colitis, sigmoid volvulus is rare in the pediatric literature with less than 100 cases reported over the last 50 years [12,13]. Sigmoid volvulus occurs due to the twisting of a redundant portion of the sigmoid colon on its mesentery, which may lead to obstruction and colonic ischemia [14]. There is some discussion that underlying constipation (i.e., Hirschsprung disease), elongated mesentery, neurological disorders, or prior abdominal surgery may predispose a patient to sigmoid volvulus. However, there are no reported cases associated with rectal seed bezoars [13,15,16]. One pediatric case report describes a jejunal trichobezoar resulting in a small bowel volvulus, and it is conceivable that the mechanism they propose, where the large weight of the bezoar displaced bowel loops and initiated rotation of the mesentery, was also present in this case of sigmoid volvulus [17]. Unlike in adult patients, there are no current consensus guidelines for the management of pediatric patients with sigmoid volvulus due to the low incidence [13,14]. Therefore, a high index of suspicion is necessary to promptly recognize and reduce the volvulus either endoscopically or surgically to prevent further complications and minimize morbidity and mortality [12].

## Conclusions

Pediatric abdominal pain is a common chief concern in pediatrics, and it is important that clinicians consider a broad differential. Rare diagnoses, such as rectal seed bezoars, are best uncovered by obtaining a comprehensive history of the present illness and maintaining a high index of suspicion. This unique case discusses previously unreported pediatric complications of rectal seed bezoars including stercoral colitis and sigmoid volvulus, and addresses the management of this rare presentation.
